# An Automated Detection System for Microaneurysms That Is Effective across Different Racial Groups

**DOI:** 10.1155/2016/4176547

**Published:** 2016-12-15

**Authors:** George Michael Saleh, James Wawrzynski, Silvestro Caputo, Tunde Peto, Lutfiah Ismail Al Turk, Su Wang, Yin Hu, Lyndon Da Cruz, Phil Smith, Hongying Lilian Tang

**Affiliations:** ^1^Moorfields Eye Hospital NHS Foundation Trust, London, UK; ^2^Department of Computing, Faculty of Engineering, University of Surrey, Guildford, UK; ^3^National Institute for Health Research Biomedical Research Centre, Moorfields Eye Hospital NHS Foundation Trust and UCL Institute of Ophthalmology, London, UK; ^4^Barking, Havering and Redbridge University Hospitals Trust, London, UK; ^5^Statistics Department, Faculty of Sciences, King Abdulaziz University, Jeddah, Saudi Arabia; ^6^Institute of Ophthalmology, UCL, London, UK

## Abstract

Patients without diabetic retinopathy (DR) represent a large proportion of the caseload seen by the DR screening service so reliable recognition of the absence of DR in digital fundus images (DFIs) is a prime focus of automated DR screening research. We investigate the use of a novel automated DR detection algorithm to assess retinal DFIs for absence of DR. A retrospective, masked, and controlled image-based study was undertaken. 17,850 DFIs of patients from six different countries were assessed for DR by the automated system and by human graders. The system's performance was compared across DFIs from the different countries/racial groups. The sensitivities for detection of DR by the automated system were Kenya 92.8%, Botswana 90.1%, Norway 93.5%, Mongolia 91.3%, China 91.9%, and UK 90.1%. The specificities were Kenya 82.7%, Botswana 83.2%, Norway 81.3%, Mongolia 82.5%, China 83.0%, and UK 79%. There was little variability in the calculated sensitivities and specificities across the six different countries involved in the study. These data suggest the possible scalability of an automated DR detection platform that enables rapid identification of patients without DR across a wide range of races.

## 1. Introduction

Patients without diabetic retinopathy (DR) represent a large proportion of the caseload seen by the DR screening service and can be time consuming to identify for human graders [1]. Microaneurysms (MAs) are the first visible sign of DR and their detection has been widely described in the literature as an important factor in identifying the onset of DR [2, 3]. As such, reliable recognition of the absence of microaneurysms initially on FFA [4] and subsequently in digital fundus images [5] (DFIs) has been a prime focus of automated DR screening research for many years.

DR detection is partly undertaken through contrast of the MA with the background field of the DFIs. The appearance of the background field in DFIs is highly variable and is influenced by many factors including the degree of pigmentation in the retinal pigment epithelium and choroid, the size of the pupil, unevenness in illumination, poor transparency of the ocular media in corneal disease and cataract, and the camera type and its settings [6]. Variability in the background field is further complicated by well-reported racial variance, for example, in the calibre of retinal blood vessels [7–9].

The effect of differing racial and image influences have not previously been evaluated in the domain of DR identification using automated screening algorithms. As the racial variance in pigmentation is known to influence the background field that automated systems use to operate it is important to know whether this will influence their performance of this important detection task [10]. Better defining these potential effects will be central to the future functionality and clinical usefulness of these platforms.

The aim of this study is to investigate the use of an automated DR detection algorithm to assess retinal DFIs and to evaluate its performance across patients of different racial backgrounds relative to a human observer.

## 2. Materials and Methods

This was a retrospective, masked, and controlled image-based study. Full Institutional Review Board approval was obtained for this study. The retrospective use of anonymized retinal images from data banks did not require ethics approval.

### 2.1. Subjects

A dataset from Moorfields Eye Hospital of digital fundal images of patients from six different countries and of varying race was retrospectively identified. In total 17,850 digital fundus images from population studies in six different countries were identified. The number of images from each country was Kenya 12,587, Botswana 500, Norway 840, Mongolia 1636, China 1079, and UK 1208. The images from Kenya and Norway originate from population screening, while those from Botswana and Mongolia originate from DR screening. The images from the UK and China originate from patients attending ophthalmic clinics: in the case of the UK, all patients had diabetes. All patients were aged 18 or over. In all cases patients were almost exclusively of the racial group that predominates in the relevant country as follows: Kenya and Botswana: African; Norway and the UK: Caucasian; China: Chinese; and Mongolia: Mongolian.

The prevalence of microaneurysms in the images as determined by human graders (the current gold standard) was as shown in Table 1.

These images were presented to the automated system described below in order to identify the eyes that did not have any evidence of microaneurysms.

### 2.2. Digital Fundus Photographs

One fovea centred digital fundus photograph was taken of each eye. The present study examined the fovea centred images. The resolution of the images varied from 3888 × 2592 pixels in Mongolia to 768 × 576 pixels in the UK.

### 2.3. The Daphne Automated Microaneurysm Detection System

The Daphne automated microaneurysm detection system assesses digital fundus images in order to detect microaneurysms. It is a noncommercialised software package newly developed in the Department of Computer Science within the Faculty of Engineering and Physical Science at the University of Surrey [11]. The aim of the system is to detect microaneurysms with high sensitivity, thus allowing any images where no microaneurysms are detected to be considered free of DR.

#### 2.3.1. System Development

The automated system was built to detect microaneurysms in a three-stage process as described below. The system was then calibrated by presenting it with 50 digital fundus photographs from the retinopathy online challenge (ROC). Each image had already been graded by humans allowing feedback on the system's performance. The results from the 50 training cases were used to fine-tune the system's parameters. The system was then tested using unseen retinal photographs from the datasets described above.

#### 2.3.2. Technical Specifications

The processing time per image on a computer equipped with an Intel Core i5 processor running at 2.2 GHz was one minute. The method is currently implemented using MATLAB. This software is currently only available for research.

#### 2.3.3. Three-Stage Process of Microaneurysm Detection

*(1) Image Preprocessing*. Images are assessed for quality and those that do not meet the required quality standard are excluded from further analysis. Image quality is acceptable if the retinal vasculature could be visualised and they were correctly centred on the disc or fovea as appropriate. Images of varying resolutions are then automatically resized and cropped to a standard size and resolution.

Colour digital fundus photographs meeting the quality criteria are passed through a red-free filter in order to render microaneurysms and other vascular features as dark lesions. A Gaussian filter is then applied to the red-free image in order to enhance small and dark structures, which include microaneurysms. Bright lesions (such as cotton wool spots and exudates) are removed from the image using a shade correction algorithm [12] in order to prevent small darker gaps between two adjacent bright lesions being confused for a microaneurysm.

*(2) Extraction of All Potential Microaneurysm Candidate Lesions*. All lesions in the preprocessed photograph that are possible microaneurysms are detected. This is achieved by passing the image through a multilayered dark object filtering method [11, 13] which was set to search for dark lesions approximately seven pixels in diameter. This step has a high sensitivity but low specificity for microaneurysms.

**Figure 1:**
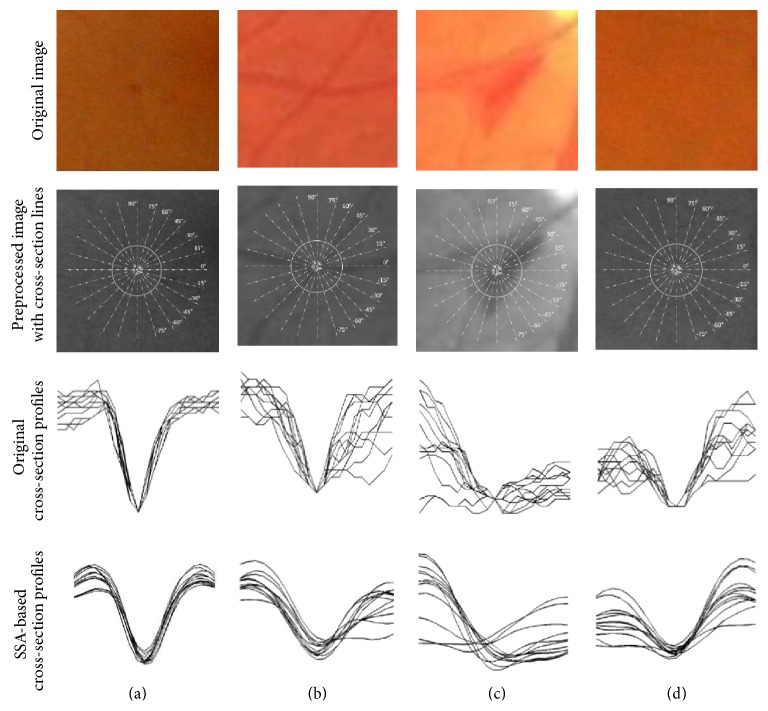
SSA cross-section profiles of different objects. (a) MA, (b) blood vessels crossing, (c) haemorrhage (an elongated non-MA structure), and (d) a retinal background. The white circles in the middle of the images indicate the actual cross-section scanning regions with 31-pixel diameter.

*(3) Selection of True Microaneurysms from Candidate Pool*. The pool of candidate lesions (possible microaneurysms) is further processed to separate the true positives from the false positives. The candidate lesions are analysed using singular spectrum analysis (SSA) [14]. SSA involves profiling the intensity of the candidates by generating several cross-sectional profiles through the candidate in different orientations. Microaneurysms tend to differ from other dark lesions such as haemorrhages or small blood vessels in that they display a high level of rotational symmetry. Therefore, a candidate was determined to be a microaneurysm when there was a high level of similarity between all cross-sectional intensity profiles at all orientations. This is demonstrated graphically in Figure 1.

### 2.4. Classification of the Images by Trained Human Graders

A human grader classified the fundus photographs into two groups based on the presence or absence of DR according to the UK national screening guidelines for diabetic retinopathy. A second human grader reclassified a minimum of 10% of randomly selected images for quality control. Human graders were based at the Reading Centre at Moorfields Eye Hospital, London, UK, and at the Department of Computer Science, University of Surrey, UK. All human graders had undergone standard training as required to be a grader for the UK's DR screening programme.

In cases where there was disagreement between machine and human grading, a senior human grader decided the appropriate grading.

### 2.5. Statistical Analysis

The sensitivity and specificity of the automated system's ability to detect DR were analysed for each of the populations studied, taking the human graders' results as the gold standard. The sensitivity and specificity values and their standard deviations across different races were analysed using R Studio software, R consortium, Boston, MA, USA [15]. Standardised positive and negative predictive values of the automated system were also calculated for each country based on the known prevalence of DR amongst patients with diabetes within the relevant racial group, as reported by Yau et al. 2012 [16]: 27% for China and Mongolia, 56% for Kenya and Botswana, and 47% for Norway and the UK.

## 3. Results

The sensitivities, defined as the proportion of patients classified as having microaneurysms by human graders that were correctly identified by the automated system, were as follows: Kenya 92.8%, Botswana 90.1%, Norway 93.5%, Mongolia 91.3%, China 91.9%, and UK 90.1%. The standard deviation in the sensitivity values across the six countries was 1.3%.

The specificities, defined as to the proportion of patients classified as not having microaneurysms by human graders that were correctly identified as such by the automated system, were Kenya 82.7%, Botswana 83.2%, Norway 81.3%, Mongolia 82.5%, China 83.0%, and UK 79.0%. The standard deviation in the specificity values across the six countries was 1.5%.

The positive predictive values were Kenya 87%, Botswana 87%, Norway 82%, Mongolia 66%, China 67%, and UK 79%. The negative predictive values were Kenya 90%, Botswana 87%, Norway 93%, Mongolia 96%, China 97%, and UK 90%.

## 4. Discussion

The prevalence of diabetes is increasing worldwide due to population growth and increasing rates of obesity and physical inactivity. Projections suggest that the prevalence of diabetes in the USA will rise from 14% (in 2010) to between 25% and 28% by 2050 [12] and the worldwide prevalence will rise from 2.8% (in 2000) to 4.4% by 2030, with an expected worldwide population of 366 million people with diabetes. The most significant increase over the three decades is expected to involve the Middle East (+163%), Sub-Saharan Africa (+161%), China (+104%), and India (+151%) [17]. 2011 census data shows a rise in the English and Welsh population of people of middle eastern, Sub-Saharan African, and Indian origin, which may further accelerate the rise in the prevalence of diabetes in the UK [18].

DR screening programmes have been recognized as an effective method for disease detection at the earliest stage of the disease to potentially prevent permanent visual loss. However, due to a rapidly expanding population of patients with diabetes, the screening programme will require significant annual increases in investment, along with the recruitment and training of an increasing number of qualified human graders. In order to alleviate this problem, several attempts have been made to develop an automated system to assist human graders [19].

The specificity of our automated system remained consistent across all the racial groups examined with standard deviation of only 1.3% and results ranging from 83.2% in Botswana to 81.3% in Norway confirming its ability to accurately detect normal images irrespective of the race of the patient. Its sensitivity ranged from 91.1% in Botswana and the UK to 93.5% in Norway, with a standard deviation of 1.5%, demonstrating that it does not miss DR in the vast majority of cases and was again consistent across the different racial groups studied. In some cases, DR that was missed by human graders was detected by the system. In such cases a human grader reevaluated the images.

The standardised negative predictive value of the system was highly consistent across all countries varying from 87% in Botswana to 97% in China, suggesting that negative results (no MAs) are highly likely to be accurate. Conversely the positive predictive value ranged between 66% in Mongolia and 87% in Kenya and Botswana, suggesting that positive results (presence of MAs) would require further human grading. Thus, the value of this system lies chiefly in its ability to rapidly and reliably identify the absence of MAs, a task which may take a human grader much longer than identifying MAs when they are present. Given that the majority of patients attending screening do not have DR, the use of such a system has the potential to vastly reduce the number of images requiring human grading.

One key limitation in this study is that the performance of the system was evaluated only relative to human grading of the same images rather than additional modalities such as other automated systems or FFA. Therefore, it is only possible to conclude that the system tends to give very similar results to humans when grading DFIs without retinopathy across a wide range of racial groups. It cannot account for any possible systematic errors in grading if humans are also susceptible to those same mistakes. In addition, although the system was built to detect microaneurysms, it is impossible to compare which exact MAs the automated system detected compared to humans.

The previously reported structural and anatomical retinal differences amongst different racial groups including macular pigment distribution [10], the presence of the parafoveal ring, and the retinal microvascular calibre variation [7, 8] along with clinically visible differences in pigmentation do not appear to have impacted significantly on the performance of this automated system, which performed well across a wide range of different racial groups. This represents a significant advantage of the Daphne system that has not been specifically tested for in previous automated systems. Similarly, differences in image quality and illumination have not had a significant effect on the automated detection of DR.

## 5. Conclusions

The data presented here suggests the possible scalability of an automated microaneurysm detection platform that enables rapid identification of patients without DR across a wide range of races.

## Figures and Tables

**Table 1:** Prevalence of microaneurysms within each studied image dataset as determined by human graders.

	Prevalence of microaneurysms as detected by human graders
Kenya	9.2%
Botswana	30.4%
Norway	9.6%
Mongolia	11.3%
China	8.2%
UK	11.7%
